# Severe Cutaneous Adverse Drug Reactions at a Tertiary Care Center in Saudi Arabia

**DOI:** 10.1155/2023/8928198

**Published:** 2023-05-10

**Authors:** Mohammed I. AlJasser

**Affiliations:** ^1^College of Medicine, King Saud Bin Abdulaziz University for Health Sciences, Riyadh, Saudi Arabia; ^2^King Abdullah International Medical Research Center, Riyadh, Saudi Arabia; ^3^Division of Dermatology, Ministry of National Guard Health Affairs, Riyadh, Saudi Arabia

## Abstract

**Background:**

Severe cutaneous adverse drug reactions (SCARs), although rare, are known to be associated with significant morbidity and mortality. SCARs include drug reaction with eosinophilia and systemic symptoms (DRESS), Stevens–Johnson syndrome/toxic epidermal necrolysis (SJS/TEN), and acute generalized exanthematous pustulosis (AGEP). Studies on SCARs are limited in Saudi Arabia. This study aims to characterize SCARs at a tertiary care center in Saudi Arabia.

**Methods:**

A cross-sectional study was conducted at King Abdulaziz Medical City, Riyadh, Saudi Arabia. All inpatient and emergency department consultations to dermatology were electronically reviewed during the period from January 2016 to December 2020. All patients who developed an adverse cutaneous drug reaction were enrolled. Detailed analysis was performed only for SCARs. The culprit medication was determined based on the latency period, history of previous intake of the medication, and drug notoriety.

**Results:**

There were 3050 hospital consultations to dermatology during the study period. Cutaneous adverse drug reactions constituted 253 (8.3%) cases. A total of 41 patients with SCARs were identified, accounting for 16.2% of all cutaneous drug reactions. Antibiotics and anticonvulsants were the most common causative drug groups accounting for 28 (68.3%) and 9 (22%) cases, respectively. DRESS was the most common SCAR. The latency period was the longest for DRESS and shortest for AGEP. Vancomycin was responsible for approximately a third of DRESS cases. Piperacillin/tazobactam was the most common cause for SJS/TEN and AGEP. The majority of drugs causing AGEP were antibiotics. The mortality rate was the highest in SJS/TEN (5/11 (45.5%)), followed by DRESS (1/23 (4.4%)) and AGEP (1/7 (14.3%)).

**Conclusion:**

SCARs are rare in Saudis. DRESS appears to be the most common SCAR in our region. Vancomycin is responsible for most cases of DRESS. SJS/TEN had the highest mortality rate. More studies are required to further characterize SCARs in Saudi Arabia and Arabian Gulf countries. More importantly, thorough studies of HLA associations and lymphocyte transformation tests among Arabs with SCARs are likely to further improve patient care in the Arabian Gulf region.

## 1. Introduction

Cutaneous adverse drug reactions (CARs) are common and occur mainly due to systemic medications. CARs are divided based on severity into two types: nonsevere CARs and severe cutaneous adverse drug reactions (SCARs). Most CARs are not severe in nature. Examples of nonsevere CARs include maculopapular drug eruption, urticaria, fixed drug eruption, drug-induced immunobullous disorders, lichenoid drug eruption, vasculitis, and photoallergic dermatitis [1, 2]. In a study of 137 Saudi adults with CARs, 35% were in the form of urticaria [3].

Severe cutaneous adverse drug reactions (SCARs), although rare, are associated with significant morbidity and mortality [4]. Therefore, it is crucial for all healthcare providers to be aware of SCARs in order to properly manage affected patients and promptly discontinue the offending medication. SCARs include drug reaction with eosinophilia and systemic symptoms (DRESS), Stevens–Johnson syndrome/toxic epidermal necrolysis (SJS/TEN), and acute generalized exanthematous pustulosis (AGEP) [4].

Patients with DRESS develop generalized erythematous papules and plaques with associated fever, eosinophilia, and liver and kidney impairment. SJS/TEN presents with severe mucous membrane erosions and separation of large skin areas. Hence, those patients are at a high risk of sepsis and electrolyte imbalance. Generalized pustules, fever, and leukocytosis are the main features of AGEP. Medication classes most commonly associated with SCARs include xanthine oxidase inhibitors (mainly allopurinol), antibiotics, and antiepileptics.

There seem to be a limited number of studies looking into SCARs in Saudi Arabia. This study aims to characterize SCARs at a tertiary care center in Saudi Arabia.

## 2. Methods

This was a cross-sectional study conducted at King Abdulaziz Medical City, Riyadh, Saudi Arabia. The study was approved by the local ethics committee (RC17/293/R). All inpatient and emergency department consultations to dermatology were electronically reviewed during the period from January 2016 to December 2020. The clinical notes in each consultation were reviewed individually in order to not miss any case of CAR.

All patients who developed an adverse cutaneous drug reaction were enrolled. Detailed analysis was performed only for SCARs. In order to verify the diagnosis for each SCAR, clinical presentation and diagnostic scores were used [4]. For the DRESS RegiSCAR and AGEP EuroSCAR scores, only “probable” and “definite” cases were included. SCORTEN was calculated for SJS/TEN cases. The culprit medication was determined based on the latency period, history of previous intake of the medication, and drug notoriety. The latency period (days) is from the initiation of a drug to the development of SCAR. Other collected variables included cutaneous and systemic manifestations, laboratory abnormalities, treatment, and complications (mainly death).

Statistical analysis was performed with JMP statistical software version 17. Categorical variables were presented as frequencies and percentages. Numerical variables were presented as the mean with standard deviation (SD) or median with an interquartile range (IQR). A comparison of numerical variables between the two groups was performed by a *t*-test. A *P* value of <0.05 was considered statistically significant.

## 3. Results

There were 3050 hospital consultations to dermatology during the study period. CARs constituted 253 (8.3%) cases. Morbilliform drug eruption was the most common CAR (180 (71.1%)). A total of 41 patients with SCARs were identified, accounting for 16.2% of all cutaneous drug reactions. Twenty-one (51.2%) were female. The mean (SD) age was 48.1 (25.8) years (median 47; IQR 47.5), and the majority were adults (35 (85.37%)). Antibiotics and anticonvulsants were the most common causative drug groups accounting for 28 (68.3%) and 9 (22%) cases, respectively. The mean (SD) latency period was 13.6 (9.9) days (median 11; IQR 16.5) from the initiation of the drug to the development of eruption.

DRESS was the most common SCAR (*n* = 23, 56.1%), followed by SJS-TEN (*n* = 11, 26.8%), and AGEP (*n* = 7, 17.1%). Among SJS/TEN cases, TEN (*n* = 5) and SJS (*n* = 4) subtypes were the most common. There was one case of SJS-TEN and DRESS-TEN overlap.  Table 1 summarizes the clinical and laboratory characteristics of all patients. Gender distribution was similar in all SCARs. SJS/TEN occurred in older patients. The latency period was the longest for DRESS and shortest for AGEP. Antibiotics were the main cause of all SCARs followed by anticonvulsants.

The prevalent features of DRESS were high-grade fever, facial edema, eosinophilia, atypical lymphocytes, and elevated liver enzymes. Abnormal renal function was found in a quarter of patients with DRESS. The mucous membranes were affected in the majority of patients with SJS/TEN. Interestingly, eosinophilia and atypical lymphocytes were noted in 36.4% and 63.6% of patients, respectively. High-grade fever was present in more than half of AGEP cases. All patients with AGEP had leukocytosis.

Culprit medications for each type of SCARs are listed in Table 2. Vancomycin was responsible for approximately a third of DRESS cases. The second most common culprit was phenytoin followed by piperacillin/tazobactam, meropenem, and lamotrigine. Piperacillin/tazobactam was the most common cause for SJS/TEN and AGEP. Meropenem and phenytoin were the next most common culprits for SJS/TEN (27.3% and 18.2%, respectively). The majority of drugs causing AGEP were antibiotics.

The suspected causative drug was discontinued in all cases (Table 3). All SJS/TEN patients received symptomatic and supportive care usually in collaboration with plastic surgery. Topical corticosteroids were mostly used for patients with DRESS or AGEP. The majority of DRESS cases were managed with systemic corticosteroids. Systemic corticosteroids and intravenous immunoglobulin (IVIG) were the most common therapies used in SJS/TEN. Only a few SJS/TEN patients were treated with cyclosporine or etanercept.

Complications occurred in 9 (22%) patients. The mortality rate was the highest in SJS/TEN (5/11 (45.5%)), followed by AGEP (1/7 (14.3%)) and DRESS (1/23 (4.4%)). The cause of death was sepsis in all cases. The mean (SD) SCORTEN was 4 (1.5). SCORTEN was higher in patients who died (4.6 (1.3) vs. 3.5 (1.6)), but this was not statistically significant (*P*=0.253). Of the 5 patients with SJS/TEN who died, 3 had IVIG, 2 had systemic corticosteroids, and 1 had etanercept. Neither of the 2 patients who received cyclosporine died from SJS/TEN. Hypopigmentation and nail loss were documented only for the patient with DRESS-TEN overlap. One patient with DRESS developed high levels of thyroid-stimulating hormone (TSH) 6 years after DRESS occurred.

## 4. Discussion

SCARs are rare and accounted for only 16.2% of all CARs in our patients. Variable rates were found in studies from Malaysia (15.8%) [5], China (22%) [6], India (6.9%) [7], and Brazil (34.2%, 30%) [8, 9]. Studies on CARs appear to be limited in general in the Middle East. Most studies were conducted in Turkey. SCARs were found in only 13% of 106 Turkish patients with CARs [10]. An analysis of CARs in 94 Turkish patients showed that SCARs constituted only 12% of all reactions [11]. In another Turkish study, CARs in 122 children were analyzed. Only 3% of patients had SCARs [12]. In a study of a 100 patients in Oman, SCARs comprised 10% of all cases of CARs [13].

DRESS was the most common SCAR in our study population. This finding was in agreement with studies from the UK [2] and Latin America [14]. A similar trend was noted in previous Turkish studies [10, 11]. An Iranian study on 282 patients with CARs found that SJS/TEN was the most common overall CAR affecting 43% of patients. Other types of CARs in descending frequency included maculopapular drug eruption (25%), urticaria (10%), DRESS (6.4%), fixed drug eruption (4%), and AGEP (2.8%) [15]. An Indian study showed that SJS/TEN and DRESS were the most and least common, respectively [7]. In Asia, SJS/TEN appears to be the most common followed by DRESS and AGEP [16, 17]. The predominance of DRESS in our study can be attributed to genetic differences.

Clinical features of SCARs were in accordance with those of the previous literature. Patients with DRESS usually present with high-grade fever, facial edema, and indurated erythematous papules and plaques affecting a large body surface area. Eosinophilia and atypical lymphocytes are commonly found in patients with DRESS. Liver involvement is typically more common than renal involvement [18]. SJS/TEN typically presents with predominant mucous membrane involvement and epidermal detachment [19]. AGEP classically presents with numerous pustules on the erythematous base mainly affecting flexural areas. Leukocytosis and high-grade fever are seen in the majority of patients [20].

DRESS had the longest latency period, while AGEP had the shortest one, which is similar to previous reports [7, 16]. DRESS is known to characteristically have a long latency period ranging from 3 to 8 weeks [18]. Most AGEP cases develop within 1-2 days after starting a medication [20].

Antibiotics caused most SCARs in our study followed by anticonvulsants. Dibek Misirlioglu et al. [21] described SCARs in 58 pediatric patients. The two most common causative drug classes were antibiotics and anticonvulsants. A similar pattern was seen in Indians [7]. Strong associations of SCARs with HLA were reported and depend on ethnicity and drug types. In East Asians, allopurinol and anticonvulsants appear to be the most common culprits for DRESS and SJS/TEN due to HLA-based susceptibility [16, 19, 22]. As a preventative measure, the US Food and Drug Administration (FDA) recommends performing genetic testing in East Asians before taking carbamazepine [19]. Antibiotics were responsible for the majority of SJS/TEN cases in our study (72.8%) followed by anticonvulsants. This pattern was similar to that of a recent study on SJS/TEN in 10 Saudi patients [23]. In AGEP, antibiotics generally appear to be responsible for most cases which are similar in our cases [4]. Genetic susceptibility seems to be less known for AGEP although there is some evidence implicating the IL-36RN gene [20].

Vancomycin was strikingly responsible for approximately a third of DRESS cases. Vancomycin-induced DRESS is commonly reported [24, 25]. A strong HLA − A*∗*32 : 01 association was previously reported in patients with European background living in North America who developed vancomycin-induced DRESS [26]. In a Spanish study on 14 patients with DRESS, HLA − A*∗*32 : 01 was found in 36% of cases [27]. Furthermore, the lymphocyte transformation test (LTT) showed high sensitivity and specificity in cases of vancomycin-induced DRESS.

The most important step in treating SCARs is to stop the culprit medication which was carried out in all patients. Performing that as early as possible might reduce mortality [28]. Supportive care was received by all patients with SJS/TEN in collaboration with plastic surgery. This is the most important step in treating SJS/TEN after drug withdrawal [29]. All patients with DRESS received topical corticosteroids, and the majority also had systemic corticosteroids. In a French study of 38 patients with DRESS, 66% were treated with only topical corticosteroids and none of the patients died. Only severe cases were treated with systemic corticosteroids [30]. Systemic corticosteroids and IVIG were the most common therapies used in SJS/TEN in our patients. Only one patient was treated with etanercept. Etanercept has shown promise in treating SJS/TEN, but more studies are needed [31]. Cases of AGEP were mostly treated with topical corticosteroids, and systemic corticosteroids are usually not required [4].

The mortality rate was the highest in SJS/TEN (5 (45.5%)), and SCORTEN was higher in those who died. The mean SCORTEN in patients who died was 4.6 which translates into a predicted mortality of >50% [4, 32]. One of the patients with DRESS developed high TSH approximately 6 years after the diagnosis of DRESS. Several autoimmune diseases including thyroid disease might develop months to years later [33].

Our study is limited by the small study sample and retrospective design given the rarity of SCARs. The number of SJS/TEN patients was too small to make relevant correlations between mortality and treatment modality.

## 5. Conclusion

To the best of our knowledge, this is the only study specifically evaluating SCARs in the Arabia Gulf region. SCARs are rare compared to other more common CARs. DRESS appears to be the most common in our region. Vancomycin is responsible for most cases of DRESS. Treatment of DRESS should start with only topical corticosteroids unless there is severe involvement requiring systemic corticosteroids. More studies are required to further characterize SCARs in Saudi Arabia and Arabian Gulf countries. More importantly, thorough studies of HLA associations among Arabs with SCARs (e.g., vancomycin-induced DRESS) should be performed in order to prevent them from occurring. Research and clinical application of LTT in SCARs will likely further improve patient care in the Arabian Gulf region.

## Figures and Tables

**Table 1:** Clinical and laboratory characteristics of patients with severe cutaneous adverse drug reaction (*n* = 41).

		DRESS		SJS/TEN		AGEP	
		*n*	%	*n*	%	*n*	%
*Gender*							
Female		11	47.8	6	54.6	4	57.1
Male		12	52.2	5	45.5	3	42.9
Age (years)	Mean (SD)	41.0 (25.4)		63.5 (24.5)		47.3 (21.2)	
	Median (IQR)	41 (35)		71 (12)		40 (32)	
*Age group*							
Adult		19	82.6	10	90.9	6	85.7
Pediatric		4	17.4	1	9.1	1	14.3
*Culprit medication class*							
Antibiotics		14	60.9	8	72.7	6	85.7
Anticonvulsants		7	30.4	2	18.2	0	0
Miscellaneous		2	8.7	1	9.1	1	14.3
Latency period (days)	Mean (SD)	17.3 (10.1)		12.1 (7.5)		3.9 (4.0)	
	Median (IQR)	15 (16)		10 (13)		2 (5)	
Fever ≥38.5°C		18	78.3	3	27.3	4	57.1
Facial edema		15	65.2	3	27.3	0	0
Mucosal involvement		1	4.4	8	72.7	0	0
Oral involvement		1	4.4	8	72.7	0	0
Eye involvement		0	0	5	45.5	0	0
Genital involvement		0	0	5	45.5	0	0
Enlarged lymph nodes		3	13	0	0	0	0
Eosinophilia		22	95.7	4	36.4	2	28.6
Eosinophilia grade 1^*∗*^		5	21.7	2	18.2	2	28.6
Eosinophilia grade 2		17	73.9	2	18.2	0	0
Atypical lymphocytes		22	95.7	7	63.6	1	14.3
Leukocytosis		15	65.2	5	45.5	7	100
Leukopenia		2	8.7	3	27.3	0	0
Thrombocytosis		7	30.4	2	18.2	2	28.6
Thrombocytopenia		4	17.4	8	72.7	1	14.3
Abnormal liver function test		18	78.3	5	45.5	1	14.3
Abnormal renal function test		6	26.1	1	9.1	0	0
Lung involvement		1	4.4	0	0	0	0

AGEP, acute generalized exanthematous pustulosis; DRESS, drug reaction with eosinophilia and systemic symptoms; IQR, interquartile range; SD, standard deviation; SJS/TEN, Stevens–Johnson syndrome/toxic epidermal necrolysis. ^*∗*^Grade 1 eosinophilia 700–1499/*μ*L; grade 2 eosinophilia ≥1500/*μ*L.

**Table 2:** Culprit medications for each type of severe cutaneous adverse drug reaction (*n* = 43).

	DRESS		SJS/TEN		AGEP	
	*n*	%	*n*	%	*n*	%
Vancomycin	7	**30.4**				
Piperacillin/tazobactam	2	8.7	4	**36.4**	3	**42.9**
Meropenem	2	8.7	3	27.3	1	14.3
Sulfamethoxazole/trimethoprim	1	4.4	1	9.1		
Amoxicillin					1	14.3
Ceftriaxone	1	4.4				
Ciprofloxacin	1	4.4				
Azithromycin					1	14.3
Metronidazole					1	14.3
Phenytoin	4	17.4	2	18.2		
Phenobarbital			1	9.1		
Carbamazepine	1	4.4				
Lamotrigine	2	8.7				
Allopurinol	1	4.4				
Esomeprazole	1	4.4				
Furosemide					1	14.3
Pembrolizumab			1	9.1		

AGEP, acute generalized exanthematous pustulosis; DRESS, drug reaction with eosinophilia and systemic symptoms; SJS/TEN, Stevens–Johnson syndrome/toxic epidermal necrolysis. ^*∗*^Two patents had two culprit medications. Phenytoin and phenobarbital in the patient with DRESS-TEN overlap. Piperacillin/tazobactam and azithromycin in a patient with AGEP. ^*∗∗*^The percentage of the most common culprit medication for each severe cutaneous adverse drug reaction is in bold.

**Table 3:** Treatment of the different types of severe cutaneous adverse drug reaction.

	DRESS		SJS/TEN		AGEP	
	*n*	%	*n*	%	*n*	%
Discontinue culprit medication	23	100	11	100	7	100
Symptomatic and supportive care	0	0	11	100	0	0
Topical corticosteroids	23	100	3	27.3	6	85.7
Systemic antihistamines	8	34.8	1	9.1	2	28.6
Systemic corticosteroids	18	78.3	4	36.4	2	28.6
Cyclosporine	0	0	2	18.2	0	0
Etanercept	0	0	1	9.1	0	0
IVIG	0	0	4	36.4	0	0

AGEP, acute generalized exanthematous pustulosis; DRESS, drug reaction with eosinophilia and systemic symptoms; IVIG, intravenous immunoglobulin; SJS/TEN, Stevens–Johnson syndrome/toxic epidermal necrolysis.

## Data Availability

Data are available on request.
